# Human LH and hCG stimulate differently the early signalling pathways but result in equal testosterone synthesis in mouse Leydig cells in vitro

**DOI:** 10.1186/s12958-016-0224-3

**Published:** 2017-01-05

**Authors:** Laura Riccetti, Francesco De Pascali, Lisa Gilioli, Francesco Potì, Lavinia Beatrice Giva, Marco Marino, Simonetta Tagliavini, Tommaso Trenti, Flaminia Fanelli, Marco Mezzullo, Uberto Pagotto, Manuela Simoni, Livio Casarini

**Affiliations:** 1 Unit of Endocrinology, Department of Biomedical, Metabolic and Neural Sciences, University of Modena and Reggio Emilia, NOCSAE, via P. Giardini 1355, 41126 Modena, Italy; 2 Center for Genomic Research, University of Modena and Reggio Emilia, via G. Campi 287, 41125 Modena, Italy; 3 Department of Neurosciences, University of Parma, via Voltuno 39/E, 43125 Parma, Italy; 4 Department of Laboratory Medicine and Pathological Anatomy, Azienda USL. NOCSAE, Via P. Giardini 1355, 41126 Modena, Italy; 5 Endocrinology Unit, Department of Medical and Surgical Sciences, Centre for Applied Biomedical Research (C.R.B.A.), S. Orsola-Malpighi Hospital. Alma Mater University of Bologna, via G. Massarenti 9, I-40138 Bologna, Italy; 6 Department of Medicine, Endocrinology, Metabolism and Geriatrics, Azienda USL. NOCSAE, Via P. Giardini 1355, 41126 Modena, Italy

**Keywords:** LH, hCG, Leydig, cAMP, Testosterone, Bioassay

## Abstract

**Background:**

Human luteinizing hormone (LH) and chorionic gonadotropin (hCG) are glycoprotein hormones regulating development and reproductive functions by acting on the same receptor (LHCGR). We compared the LH and hCG activity in gonadal cells from male mouse in vitro, i.e. primary Leydig cells, which is a common tool used for gonadotropin bioassay. Murine Leydig cells are naturally expressing the murine LH receptor (mLhr), which binds human LH/hCG.

**Methods:**

Cultured Leydig cells were treated by increasing doses of recombinant LH and hCG, and cell signaling, gene expression and steroid synthesis were evaluated.

**Results:**

We found that hCG is about 10-fold more potent than LH in cAMP recruitment, and slightly but significantly more potent on cAMP-dependent Erk1/2 phosphorylation. However, no significant differences occur between LH and hCG treatments, measured as activation of downstream signals, such as Creb phosphorylation, *Stard1* gene expression and testosterone synthesis.

**Conclusions:**

These data demonstrate that the responses to human LH/hCG are only quantitatively and not qualitatively different in murine cells, at least in terms of cAMP and Erk1/2 activation, and equal in activating downstream steroidogenic events. This is at odds with what we previously described in human primary granulosa cells, where LHCGR mediates a different pattern of signaling cascades, depending on the natural ligand. This finding is relevant for gonadotropin quantification used in the official pharmacopoeia, which are based on murine, in vivo bioassay and rely on the evaluation of long-term, testosterone-dependent effects mediated by rodent receptor.

**Electronic supplementary material:**

The online version of this article (doi:10.1186/s12958-016-0224-3) contains supplementary material, which is available to authorized users.

## Background

Human luteinizing hormone (LH) and chorionic gonadotropin (hCG) are glycoprotein hormones which bind the same receptor (LHCGR), belonging to the superfamily of the G protein-coupled receptors (GPCRs) (Ascoli et al., 2002). Although they share similar molecular structures, the two hormones have distinct physiological roles [1]. LH is produced by the pituitary in a pulsatile fashion, inducing ovulation and maintenance of the corpus luteum in females. In adult males, LH regulates spermatogenesis acting on Leydig cells located in the testes, by controlling testosterone production [2, 3]. hCG is the primate-specific pregnancy hormone produced by trophoblast cells [4]. During development, maternal hCG stimulates the proliferation of the male foetus’ Leydig cells. Barely detectable amounts of pituitary hCG produced during the fertile age of both males and females were also demonstrated in circulation [5], but its role is still unknown.

### In vivo bioassays: methods for human gonadotropin quantification mediated by rodent Leydig cells

Due to the hCG action on LHCGR and the long half-life, this hormone is used instead of LH for treatment of infertility in males and for assisted reproduction in females. Pharmacological preparations are quantified by in vivo bioassays, which aim to assess the effects (e.g. organ weight) of hormones injected in a living rat or mouse [6] to evaluate gonadotropin bioactivity, thus inferring their dosage for clinical purposes [7]. Indeed, official Pharmacopoeias recognize the biological activity of LH and hCG preparations as determined by the so-called “Van Hell bioassay” [8]. Briefly, a fixed dose of gonadotropin is administered by daily subcutaneous injection in groups of ten 21–28 days old immature male rats. The animals are killed five days after the first injection and the seminal vesicles dissected out, dried and weighted to evaluate seminal vesicle weight gain (SVW). LH and hCG are calibrated against an International Standard using the Van Hell bioassay, which does not discriminate between LH and hCG bioactivity [9].

Main testicular functions, e.g. spermatogenesis, are critically depending on testosterone in mice and rats [2]. The steroid hormone is produced by Leydig cells expressing the murine LH receptor (mLhr), upon LH stimulation. Therefore, SVW evaluated by rat/mouse bioassays may be the representation of the gonadotropins’ steroidogenic potential triggered by heterologous receptors, not providing the full spectrum of LH- and hCG-specific, *non*-steroidogenic functions exerted by these molecules in the reproductive endocrine *milieu* of women.

### LH and hCG intracellular signaling

By acting on the common receptor, LH and hCG activate several signaling cascades typically associated to rhodopsin-like GPCRs in the human as well as other mammals [10]. Hormone binding to LHCGR triggers the activation of adenylyl cyclase through Gαs protein, resulting in intracellular cyclic adenosine-monophosphate (cAMP) increase, in downstream activation of the protein kinase A (PKA) [11, 12] which, in turn, induces extracellular signal-regulated kinases 1 and 2 (ERK1/2) phosphorylation [13]. PKA also mediates the activation of the cAMP-response element-binding protein (CREB), modulating the expression of target genes, such as the steroidogenic acute regulatory protein (*STARD1*) gene, and testosterone production. Previous studies demonstrated different interactions between the gonadotropins and their common receptor [14–17], as well as specific intracellular signaling and downstream events in goat and human granulosa lutein cells in vitro [18–20]. Especially, hCG has higher cAMP-mediated steroidogenic and pro-apoptotic potential than LH, which, in turn is more effective in activating anti-apoptotic and proliferative events *via* ERK1/2 and AKT-pathways in vitro, while similar kinetics of intracellular cAMP reversibility were found in mouse Leydig tumor cells (MLTC-1) stimulated by the two human gonadotropins [21]. However, an in-depth comparison between the human LH- and hCG-mediated signaling in Leydig cells is still lacking.

### Aim of the study

The aim of this study is to compare the signal transduction pathways elicited by human LH and hCG in mouse Leydig cells, which are commonly used to quantify human gonadotropins for clinical purposes, using an in vitro approach. Especially, cAMP production, ERK1/2 and CREB phosphorylation (pERK1/2 and pCREB, respectively), target gene expression and testosterone synthesis were evaluated. Mouse cells are naturally expressing the murine LH receptor, which shares high, but not complete sequence identity with LHCGR, as well as binding capability to human LH and hCG [13, 22]. However, it should be taken into account that some discrepancy may be observed between human and rodent LH receptor-mediated signals, as previously observed in the monkey *Callithrix jacchus* [23]. This study may provide new insights useful for interpreting the significance of rat/mouse in vivo bioassays, i.e. the methods for unit assignment to gonadotropins used for pharmacological treatment in humans. Human LH receptor mediates both quantitatively and qualitatively LH- and hCG-dependent signals in human primary granulosa cells [18, 19], revealing that LHCGR discriminates hormone-specific events. Therefore, we challenge the concept that gonadotropins’ activity can be properly characterized by the assessment of testosterone-dependent endpoints.

## Methods

### Recombinant Gonadotropins

Human recombinant LH (Luveris) and hCG (Ovitrelle) were kindly provided by Merck KGaA (Darmstadt, Germany).

### Leydig cells isolation and culture

Leydig cells were collected from 3–5 months-old C57BL6 mice following a validated protocol [24], under the permission of the local Animal Ethics Committee and current animal protection laws. Briefly, testes were mechanically dissociated and subjected to 20 mg/ml collagenase (Sigma-Aldrich, St. Louis, MO, USA) treatment by gentle shacking at 37 °C for 20 min. Cell suspension was filtered by a 100 μm Nylon mesh and Leydig cells were isolated by 0–100% v/v Percoll linear density gradient (GE Healthcare, Little Chalfont, UK) through centrifugation (800 x g; 45 min). Purity of the cell preparations was verified by 3β-hydroxysteroid dehydrogenase (3βHSD) assay [25]. Depending on the endpoint measured, Leydig cells were seeded in multi-well plates, in pH = 7.4 minimal essential medium (MEM) (Gibco, Thermo Fisher Scientific, Waltham, MA, USA), supplemented with 0.07% serum albumin (Sigma-Aldrich), 100 U/ml penicillin, 50 μg/ml streptomycin and 25 mM Hepes (Gibco). Cells were maintained two days in an incubator at 37 °C and 5% CO_2_ before stimulation.

### cAMP stimulation protocol and measurement

To evaluate cAMP accumulation upon LH or hCG stimulation, a validated protocol was followed [26]. Briefly, Leydig cells were seeded in 24-well plates (5 x 10^4^ cells/well) and cultured 2 days before stimulation. Cells were treated with increasing doses of LH or hCG (1 pM-100 nM range), in the presence of 500 μM of phosphodiesterases inhibitor 3-isobutyl-1-methylxanthine (IBMX) (#I5879, Sigma-Aldrich) [27]. Cells stimulated by 1 μg/ml cholera toxin (CTX) (Sigma-Aldrich) [28] were used as positive control, while unstimulated cells served as negative control (basal condition). After 3 h of incubation, samples were frozen. Total cAMP levels were evaluated using the Cyclic AMP Direct EIA kit (Arbor Assays, Ann Arbor, MI, USA), following the supplier’s instructions and signals were measured by a Victor3 multilabel plate reader (PerkinElmer Inc., Waltham, MA, USA). Data were entered into a curve fitting software and represented using a log regression analysis, as previously described [29].

### Western blot analysis

pErk1/2 and pCreb levels induced by LH or hCG treatment were analyzed by Western blotting, as previously described [18, 19, 29, 30]. Briefly, Leydig cells were seeded in 96-well plates (1 x 10^5^ cells/well) and treated 15 min by increasing doses of LH or hCG (1 pM-100 nM range). 10 μM of the mitogen-activated protein kinases (Mek) inhibitor U0126 (#U120, Sigma-Aldrich), or 10 μM of the PKA inhibitor H-89 (#B1427, Sigma-Aldrich) [31] were also used where appropriate. Cells were immediately lysed for protein extraction in 4 °C-cold RIPA buffer added with PhosStop phosphatase inhibitor cocktail and protease inhibitor cocktail (Roche, Basel, Switzerland). pErk1/2 and pCreb activation were evaluated by 12% SDS-PAGE and Western blotting, using specific antibodies (#9101 and #9198, respectively; Cell Signaling Technology Inc., Danvers, MA, USA). Total Erk1/2 served as loading control (#4695; Cell Signaling Technology Inc.). Signals were revealed by ECL chemiluminescent compound (GE HealthCare), after incubation of the membranes with a secondary anti-rabbit horseradish peroxidase-conjugated antibody (#NA9340V; GE HealthCare). Western blotting signals were acquired by the QuantityOne analysis software (Bio-Rad Laboratories Inc., Hercules, CA, USA) and semi-quantitatively evaluated by the ImageJ software (U. S. National Institutes of Health, Bethesda, MD, USA) [32].

### Stimulation for gene expression analysis and evaluation by Real-time PCR

The hCG and LH 80% effective doses (EC_80s_) were calculated from the aforementioned cAMP dose–response curves, and were taken as the lowest, maximally activating gonadotropin concentrations (100 pM hCG and 1 nM LH), as previously described [19, 29]. EC_80s_ were used as fixed doses for the gene expression analysis.

Leydig cells were seeded in 12-well plates (1 x 10^5^ cells/well) and stimulated 12 h by LH and hCG. Samples were lysed and subjected to RNA extraction using the automated extractor EZ1 Advanced XL (Qiagen, Hilden, Germany). Equal amounts of total RNA were retro-transcribed by iScript reverse transcriptase (Bio-Rad Laboratories Inc.) according to the following protocol: 25 °C for 5 min; 42 °C for 30 min; 85 °C for 5 min. Quantitative real-time PCR was performed in triplicates using specific mouse *Stard1* gene primer sequences (*fwd*: 5′-*ACAGACTCTATGAAGAACTT*-3′; *rev*: 5′-*GACCTTGATCTCCTTGAC*-3′) and settings: 95.0 °C for 30 s; 95.0 °C for 3 s - 45 cycles; 57.0 °C for 30 s. Normalized gene over the mouse hypoxanthine phosphoribosyltransferase (*Hprt*) gene expression (*fwd*: 5′-*TTGCTCGAGATGTCATGAAGGA*-3′; *rev*: 5′-*AGCAGGTCAGCAAAGAACTTATAG*-3′) was evaluated using the 2^-ΔΔCt^ method [33]. Primer sequences were designed and validated as previously described [18, 19, 29].

### Testosterone stimulation protocol and measurement

Leydig cells were seeded in 12-well plates (1 x 10^5^ cells/well) and treated by increasing doses of LH and hCG (1 pM-100 nM range), in the presence of 500 μM IBMX. U0126 or H-89 were used where appropriate. The stimulations were blocked by immediate freezing of the samples after 24 h, then total testosterone was measured in the media by an immunoassay analyser (ARCHITECT 2nd Generation Testosterone system; Abbot Diagnostics, Abbott Park, Chicago, IL, USA).

### Alignment of LH receptor amino acid sequences

The amino acid sequences of mouse, rat (rLhr) and human LH receptor were aligned by UniProt on-line tool (http://www.uniprot.org/align/) using default settings. The percentage of sequence identity among LHCGR, mLhr and rLhr were performed using the National Center for Biotechnology Information (NCBI) BLAST tool (http://blast.ncbi.nlm.nih.gov/Blast.cgi).

### Statistical analysis

cAMP and testosterone dose–response data were represented as total levels over basal, or normalized as percentage of the maximal response. pErk1/2 and pCreb semi-quantitative signals were normalized over total ERK and expressed in relative units. Data from gene expression analysis were represented as relative expression. All data were plotted as means ± standard error of mean (SEM). Mann-Whitney’s *U*-tests, two-way ANOVA followed by Bonferroni post-test or *non*-linear regressions were performed as appropriate. Differences were considered significant for *P* < 0.05. Statistical analysis was performed using the GraphPad Prism software (GraphPad Software Inc., San Diego, CA, USA).

## Results

### Evaluation of cAMP production

The Leydig cells isolation protocol was validated by the 3βHSD assay [24, 25], resulting in more than 95% purity.

**Fig. 1:**
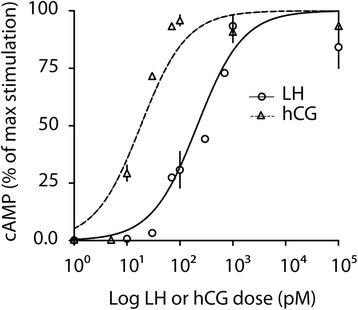
Comparison between LH- and hCG-induced cAMP production by dose–response experiment. Murine primary Leydig cells were stimulated by increasing doses of hCG and LH, in the presence of 500 μM IBMX. Total cAMP was measured after 3 h of incubation and cAMP levels were normalized as percentage of the maximal response. All the results are represented as means ± SEM in a logarithmic X-axis, then *non*-linear regressions were plotted. The EC_50_ cAMP values were compared (LH EC_50_ = 192 ± 53.96 pM; hCG EC_50_ = 18.64 ± 10.14 pM; means ± SEM; Mann-Whitney’s *U*-test; *P* = 0.0286; *n* = 4)

LH- and hCG-induced total cAMP productions were compared in murine primary Leydig cells by dose–response experiments, in the presence of IBMX, and evaluated by ELISA (Fig. 1). Although cAMP absolute levels suggest overall low response to human gonadotropin (Additional file 1), the treatments resulted in hormone-specific dose–response curves and significantly different half maximal effective concentrations (EC_50_) (LH EC_50_ = 192 ± 53.96 pM; hCG EC_50_ = 18.64 ± 10.14 pM; means ± SEM; Mann-Whitney’s *U*-test; *P* = 0.0286; *n* = 4). This reveals an approximately 10-fold higher potency of hCG *versus* LH, in terms of cAMP production, in spite of the similar cAMP *plateau* levels achieved upon 100 nM LH or hCG treatment.

### Analysis of ERK1/2 and CREB phosphorylation

**Fig. 2:**
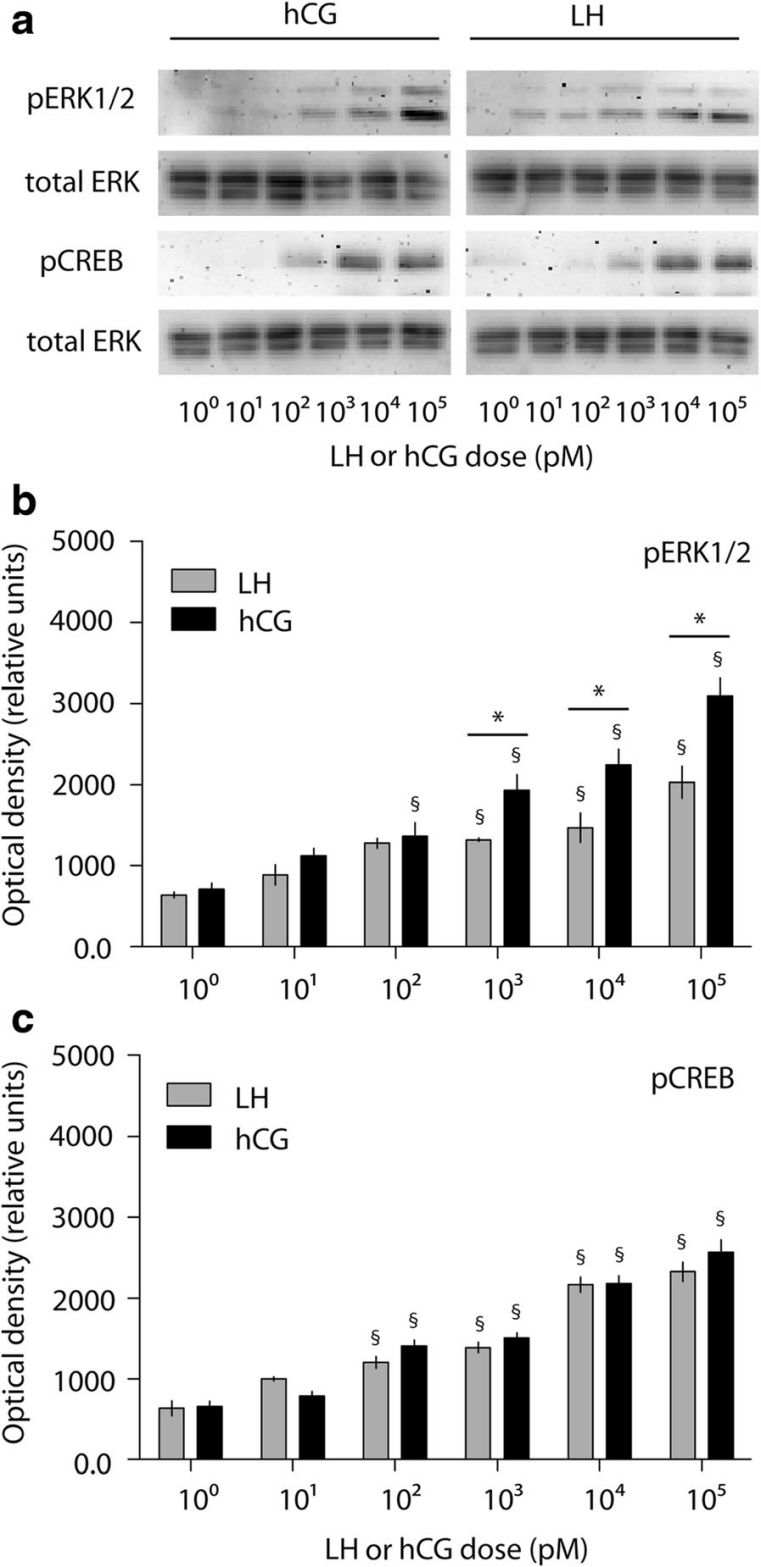
Evaluation of pErk1/2 and pCreb activation upon LH and hCG treatment. **a** Mouse Leydig cells were stimulated by increasing doses of LH and hCG. The phosphorylation of Erk1/2 and Creb was evaluated after 15 min by Western blotting (image representative of five independent experiments). **b**, **c** Densitometric analysis of pErk1/2 (**b**) and pCreb (**c**) signals. The values were normalized over total ERK and represented as means ± SEM, then statistically evaluated (§ = significant *vs* control; * = significant LH *vs* hCG; two-way ANOVA and Bonferroni post-tests; *P* < 0.05; *n* = 5)

The LH- and hCG-induced pCreb and pErk1/2 activations were compared by dose–response experiments in 15 min-stimulated primary murine Leydig cells. Cells were treated by increasing LH or hCG doses, then pCreb and pErk1/2 levels were evaluated by Western blotting and semi-quantitatively measured (Fig. 2).

Treatment by gonadotropins induced pErk1/2 activation in different concentration ranges, compared to untreated cells (LH = 100 pM-100 nM; hCG = 10 pM-100 nM; two-way Anova and Bonferroni post-test; *P* < 0.05; *n* = 5). The comparison between LH- and hCG-mediated pErk1/2 activation was also performed. We found higher Erk1/2 phosphorylation levels upon hCG *versus* LH stimulation, within the 100 pM-100 nM range (Two-way Anova and Bonferroni post-test, *P* < 0.05; *n* = 5).

pCreb activation occurs upon both LH and hCG treatment in the 10 pM-100 nM range compared to controls (two-way Anova and Bonferroni post-test; *P* < 0.05; *n* = 5), while no significant differences between LH- and hCG-induced pCreb levels were found (two-way Anova and Bonferroni post-test; *P* = 0.08; *n* = 5), not reflecting the hormone-specific differences observed in total cAMP production.

### Gene expression

**Fig. 3:**
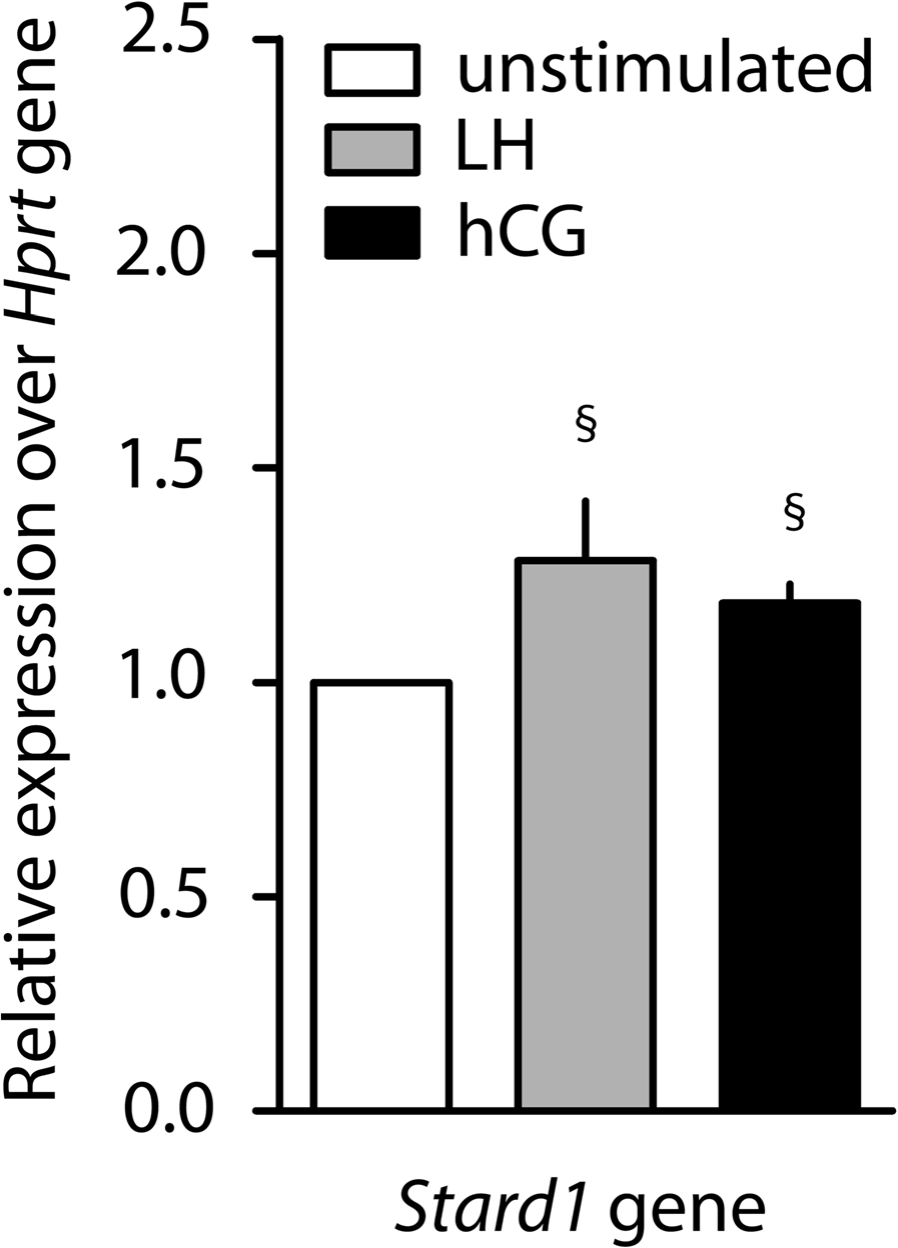
*Stard1* gene expression analysis. The expression of *Stard1* gene was evaluated in 12 h EC_80_ LH- or hCG-stimulated mouse primary Leydig cells by real-time PCR. Unstimulated cells served as control. Each value was normalized over the *Hprt* gene expression and graphically represented as fold increase over unstimulated controls in relative units scale (means ± SEM). Gene expression levels were compared (§ = significant *vs* control; Mann-Whitney’s *U*-test; *P* < 0.05; *n* = 4)

The expression of the steroidogenesis-related *Stard1* gene was analyzed as a downstream effector of the cAMP/PkA-pathway. Mouse Leydig cells were maintained 12 h in the presence of the LH or hCG EC_80_ (100 pM hCG; 1 nM LH), as the lowest dose maximally activating cAMP, and gene expression was evaluated by real time PCR (Fig. 3). *Stard1* gene expression increased upon both LH and hCG treatments compared to basal (LH = 1.29 ± 0.13 relative units; hCG = 1.19 ± 0.04 relative units; Basal = 1.0 ± 0.0 relative units; means ± SEM; Mann-Whitney’s *U*-test; *P* < 0.05; *n* = 4). However, no significant differences between LH- and hCG-induced *Stard1* expression were found (Mann-Whitney’s *U*-test; *P* = 1.00; *n* = 4), reflecting its dependence on pCreb activation.

### Evaluation of steroid production

**Fig. 4:**
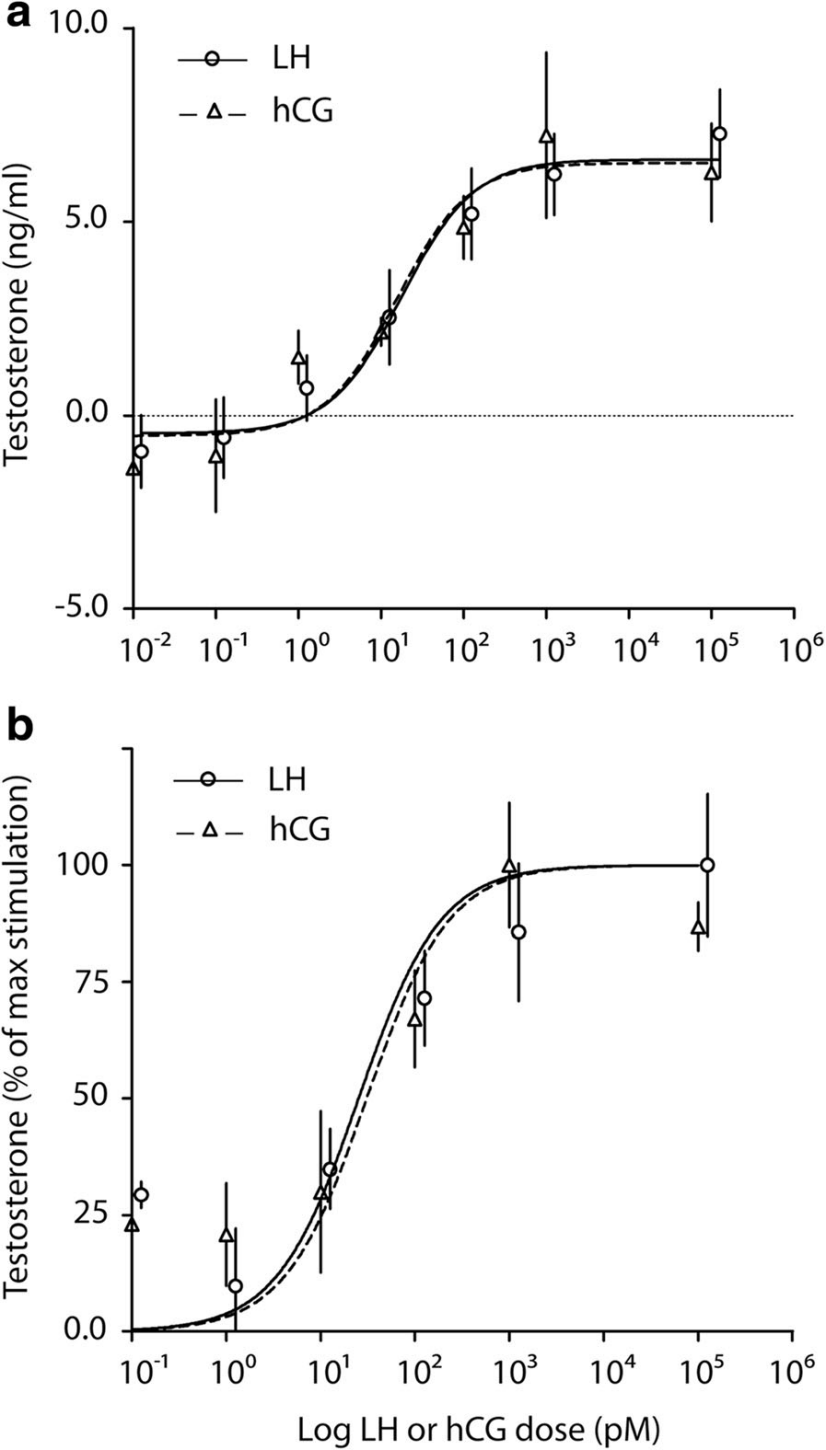
Comparison between LH- and hCG-induced testosterone production in murine primary Leydig cells. The steroid was measured in 24 h-stimulated cells by increasing LH and hCG doses, in the presence of 500 μM IBMX. **a** Total testosterone levels was measured by immuno-assay and represented as means ± SEM in ng/ml, then *non*-linear regressions were plotted. **b** Testosterone levels were normalized in percentage of the maximal response. EC_50_ values were statistically compared (hCG EC_50_ = 36.66 ± 12.25 pM; LH EC_50_ = 50.08 ± 18.03 pM; means ± SEM; Mann-Whitney’s *U*-test; *P* = 1.00; *n* = 5)

The effects of LH and hCG increasing doses (1 pM-100 nM range) on testosterone production was evaluated in murine primary Leydig cells. The steroid hormone was measured by immunoassay in the media of 24 h-stimulated cells. Since testosterone synthesis depends on intracellular cAMP, 500 μM IBMX was added 20 min before gonadotropins to optimize the steroid production [34]. No significant differences between LH- and hCG-induced testosterone production were found, resulting in similar dose–response curves and EC_50s_ (hCG EC_50_ = 36.66 ± 12.25 pM; LH EC_50_ = 50.08 ± 18.03 pM; means ± SEM; Mann-Whitney’s *U*-test; *P* = 1.00; *n* = 5). Testosterone increased similarly by either LH or hCG in a dose-dependent manner (Fig. 4), not reflecting the different LH- and hCG-induced cAMP production. The reliability of the hormone detection was confirmed performing representative measurements of steroids, i.e. 17alpha-OH-progesterone (Additional file 2), androstenedione (Additional file 3) and testosterone (Additional file 4), by liquid chromatography-mass spectrometry (LC-MS) following a previously validated method [35]. Levels of dehydroepiandrosterone (DHEA) were also measured and they are undetectable.

### pERK1/2 and pCREB activation in the presence of specific inhibitors

The results obtained so far indicate that LH and hCG treatments result in different responses measured as early signalling events occurring upon receptor activation, i.e. cAMP and pErk1/2 activation, but not in the downstream Creb phosphorylation, *Stard1* gene expression and testosterone synthesis. Taken together, these data suggest that both LH and hCG treatments result in qualitatively similar activation of balancing mechanisms orchestrated by pro- and anti-steroidogenic signals at the intracellular level, possibly mediated through cAMP and pErk1/2 [36–38], which results in equal pCreb activation. Therefore, the contribution of the cAMP/PkA- and Erk1/2-pathway in steroidogenesis was investigated by using specific inhibitors.

**Fig. 5:**
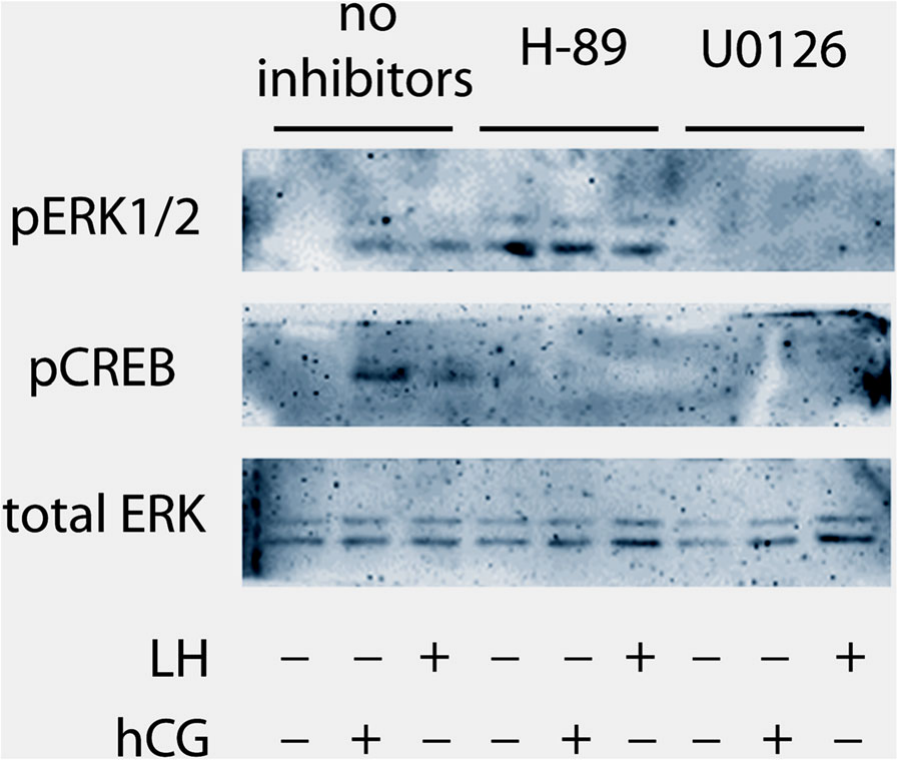
Analysis of LH/hCG-induced ERK and CREB phosphorylation in the presence/absence of specific inhibitors. Murine primary Leydig cells were stimulated 15 min by EC_80_ LH or hCG, in the presence or in the absence of PKA and MEK inhibitors (H-89 and U0126, respectively). pERK and pCREB levels were evaluated by Western blotting (image representative of three independent experiments)

pErk1/2 and pCreb activation were analysed in mouse Leydig cells stimulated by LH or hCG, in the presence of specific Mek and PkA inhibitors, i.e. U0126 and H-89, respectively. Cells were stimulated for 15 min with LH or hCG EC_80_ doses, in the presence or in the absence of U0126 or H89, and pErk1/2 and pCreb levels were evaluated by Western blotting (Fig. 5). The treatment by U0126 resulted in failure of LH/hCG-induced Creb and Erk1/2 phosphorylation. Interestingly, PkA blockade by H-89 resulted in tonic, LH/hCG-not sensitive pErk1/2 activation, as previously observed in COS-7 cells [29], and complete abolition of Creb phosphorylation.

### Steroid production in the presence of specific inhibitors

**Fig. 6:**
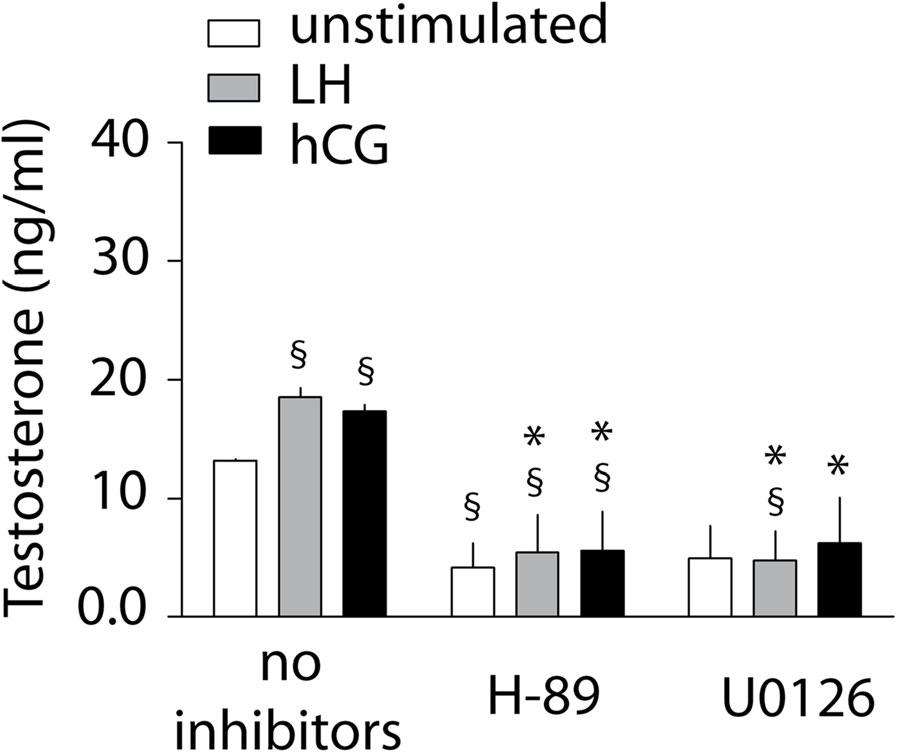
Evaluation of testosterone production upon LH or hCG stimulation in the presence or absence of specific inhibitors, in mouse Leydig cells. The cells were stimulated 24 h by EC_80_ LH and hCG, in the presence or absence of H-89 or U0126. Total testosterone levels were measured by immune-assay and represented as means ± SEM (§ = significant *vs* “unstimulated, no inhibitors”; * = significant LH/hCG *vs* corresponding LH/hCG samples without inhibitor; Mann-Whitney’s *U*-test; *P* < 0.05; *n* = 4)

Selective inhibitors were used to induce Mek and PkA blockade and testosterone production was measured in 24 h-stimulated cells by LH and hCG EC_80s_ (Fig. 6). Both treatments impaired the gonadotropin-induced testosterone synthesis, revealing that both the Erk1/2- and PkA-pathways are required for gonadotropin-stimulated steroidogenesis in Leydig cells. Interestingly, H-89 significantly decreased testosterone production even in gonadotropin-untreated mouse Leydig cells, suggesting that Pka is fundamental for the maintenance of steroid synthesis at basal level.

### Analysis of LH receptor sequences and intracellular interactions

LHCGR and rodent LH receptor amino acid sequences were compared by on-line analysis tools and are available as additional file (Additional file 5). The alignment of mLhr and rLhr revealed that the two molecules are overall structurally similar (94.2% identity), reflecting their evolutionary closeness [2, 10], while lower sequence identity was observed by mouse/rat *versus* human receptor comparison (mLhr *versus* LHCGR = 81.93%; rLhr *versus* LHCGR = 83.93%), especially at the hinge region (Table 1).

**Table 1:** Identity between human, mouse and rat LH receptors

	LHCGR *vs* mLhr	LHCGR *vs* rLhr	mLhr *vs* rLhr
Total receptor	81.9%	83.9%	94.2%
Hinge region	80.0%	81.9%	95.0%
TMD + ICD	88.0%	90.0%	93.0%

Legend: *TMD* transmembrane domain, *ICD* intracellular domain

## Discussion

In the present study, we compared the LH- and hCG-induced steroidogenic signaling, in mouse primary Leydig cells in vitro. cAMP production, pErk1/2 and pCreb activation, as well as gene expression and steroid synthesis were evaluated, demonstrating that human LH and hCG treatments result in quantitatively different, early intracellular events (i.e. cAMP and PkA-dependent pErk1/2 activation), in mouse Leydig cells. Especially, hCG has higher steroidogenic potential than LH, due to its stronger activity on cAMP/PkA-pathway activation. However, hormone-specific effects are not maintained in the downstream *Stard1* gene expression levels and steroid production in vitro, which occurs within the physiological range in mice [39, 40]. Each endpoint was evaluated at the proper time required to optimally measure its activation by practical methods, based on previous studies demonstrating their inter-dependency [18, 19, 29, 30].

### Implications for gonadotropins quantification by in vivo bioassays

This study provides new insights of relevance for the Van Hell in vivo bioassay, which aims to infer gonadotropin dosage for clinical purpose in humans [7] evaluating their bioactivity in a living rat or mouse [6]. Our results support that both human LH and hCG equally acts on testosterone production in rodent Leydig cells in vitro, not differentiating the ligand-dependent events that we demonstrated earlier in human granulosa cells [18, 19]. These cells are naturally expressing the human LH and hCG receptor, which mediates both quantitatively and qualitatively different intracellular signaling and downstream events, depending on ligand. Especially, LH-specific signals concern the predominant activation of proliferative and anti-apoptotic ERK1/2- and AKT-pathways, while treatment by hCG induced preferential cAMP/PKA-mediated steroidogenesis [18, 19]. Therefore, the in vivo bioassays might not fully appropriate for inferring the full spectrum of gonadotropin bioactivity in the human since they are based only on testosterone-dependent endpoints, detecting only one aspect of complex intracellular signaling patterns [41]. Reasonably, in vivo bioassays do not differentiate between LH- and hCG-specific activity and disregard, for instance, effects on proliferation/apoptosis important for final follicle maturation in the human ovary.

### Different early signaling mediated by LH and hCG

cAMP is an early mediator of steroidogenic signals, downstream the activation of gonadotropin receptors in steroidogenic cells [11]. Consistent with previous reports [18, 19], we found that hCG has an about 10-fold higher potency than LH in cAMP recruitment, in spite of similar *plateau* levels. This result corroborates what previously described in human granulosa cells naturally expressing the human receptor, where treatment by hCG resulted in higher cAMP/PkA pathway activation than LH in vitro, both in the presence and in the absence of FSH [18–20], suggesting a different steroidogenic potential by the two molecules. Consistently with the cAMP/PkA dependence, hCG treatment resulted in higher, dose-dependent, early (15 min) pErk1/2 activation than LH. This result mismatches what we previously obtained in human granulosa cells in vitro [18], where LH treatment resulted in higher pERK1/2 activation than hCG, revealing a qualitatively different, cAMP-independent signaling depending on the ligand. Taken together, these data suggest that human granulosa cell system discriminates between LH and hCG, while murine Leydig cell does not. Unfortunately, the direct evidence that Erk1/2 phosphorylation occurs through PkA upon LH/hCG treatment in Leydig cells cannot be demonstrated, due to the complete abolition of gonadotropin-mediated signaling by the PKA inhibitor H-89 (Fig. 5), which results in slight, tonic pErk1/2 activation.

### Similarities between LH- and hCG-mediated signaling downstream cAMP/Pka

Interestingly, we found no significant differences between LH- and hCG-induced pCreb levels in mouse primary Leydig cells, in spite a higher potency of hCG on upstream cAMP production. Thus, the two human hormones induced similar Lhr-mediated Creb phosphorylation, triggering its same, maximal activation in a dose-dependent manner. It is known that pERK1/2 activation supports both steroidogenic signals and GPCR downregulation [42], however, the levels of Erk1/2 and Creb phosphorylation induced by low LH/hCG doses (i.e. 10^1^ and 10^2^ pM) are close to the detection limits, not allowing to reliably address why gonadotropin treatments result in equal pCreb activation in the entire dose-range. Anyway, this does not correspond with that previously demonstrated in human primary granulosa cells, where pCREB achieved higher activation levels upon hCG treatment than LH, in spite of lower ERK1/2 phosphorylation [19]. Therefore, LHCGR activation results in qualitatively different, LH/hCG specific pattern of simultaneously mediated signaling cascades in human granulosa cells, different from that of murine receptor in mouse Leydig cells. Results from the gene expression analysis corroborate the pCreb activation pattern, since no different increase of LH- and hCG-induced *Stard1* gene expression levels were observed. Indeed, in murine Leydig cells, *Stard1* gene expression occurs as a pCreb-dependent event [13], although several other modulators of the steroidogenic response were described [43].

Both gonadotropins stimulate testosterone production in a dose-dependent manner, but no significant differences between hCG- and LH- induced steroid synthesis occur, as demonstrated by similar EC_50s_, corroborating the upstream steroidogenic pCreb activation and *Stard1* gene expression. PkA and Mek selective blockade by H89 and U0126 inhibitors resulted in complete testosterone synthesis suppression, demonstrating that both PkA and pErk1/2 are required for Leydig cells steroidogenesis. The multiplicity of the signaling pathways simultaneously mediated by GPCR [44] could be the cause of progressively accumulated downstream modulatory events which mask the differences observed in the early cell signaling.

### Mouse Leydig and human granulosa cells: two different endocrine systems

Our results indicate that different downstream LH- and hCG-dependent signaling characterize primary mouse Leydig and human granulosa cells [18], reflecting the specific physiological role of these systems in relation to LH-like hormone stimulations. However, qualitatively different LH/hCG-dependent intracellular signals occur between human granulosa [18] and mouse Leydig cells in vitro, suggesting diverse receptor sequences and/or intracellular *milieu* as origin of the difference.

In adult mice, Leydig cells regulate spermatogenesis through testosterone production by pituitary LH stimulation, which plays a key role in maintenance of fertility in a follicle-stimulating hormone (FSH)-independent manner [45–47]. Indeed, testosterone treatment recover the number of mature spermatids and fertility in *mLhr* knock-out mice [48, 49], as well as hCG injection in gonadotropin-deficient *hpg* mice [50]. No wide differences exist between mouse and human spermatogenesis, where testosterone is fundamental for the maturation of gametes and maintenance of fertility exerting proliferative and anti-apoptotic roles [51, 52], indicating that, in both the mammals, spermatogenesis is testosterone-dependent. Indeed, spermatogenesis can be restored also in gonadotropin-suppressed men inducing testosterone production by hCG treatment [53].

In women of fertile age, LH regulates folliculogenesis together with FSH and estrogens, and induces ovulation. Pituitary gonadotropins stimulate the concerted action of theca and granulosa cells to produce estrogens, resulting in dominant follicle growth while others undergo *atresia*. The role exerted by LH during this period seems to be unique and may not be easily replicated by hCG, since it is not equivalent to LH at the molecular level [18–20]. Even artificial cycles induced by clinical administration of gonadotropins for assisted reproduction fail to reproduce exactly the natural cycle, inducing multi-ovulations in a mono-ovulatory species. On the other hand, hCG is produced exclusively during pregnancy, except very low amount of pituitary hCG [5], when the ovarian functions are mainly focused to the production of relatively high progesterone levels instead of follicle growth.

Taken together, these data suggest that mouse/rat in vivo bioassays may not be fully informative of LH/hCG bioactivity/action, given the functional diversity of the rodent Leydig and human granulosa/theca cell systems. Further studies may evaluate the LH/hCG-mediated intracellular signals in human Leydig cells, which should provide a better in vitro system to be compared to human primary granulosa cells. However, the poor availability of human Leydig cell donors will be a challenging limitation in this regard.

### Different ligand-LH receptor features between human and mouse

It was recently reported that murine receptor is able to mediate qualitatively different response to LH and hCG, both in terms of cAMP/PKA- and β-arrestin-dependent pathways in transfected HEK293 cells, as well as downstream steroid synthesis in mLTC-1 cells endogenously expressing mLhr [54]. These data suggest that transfected/tumor cell lines may not be fully representative of the wide range of gonadotropin-mediated intracellular responses occurring in primary cells, as previously observed [30, 55]. However, these findings confirm what was previously observed in human granulosa cells [18, 19], revealing that mouse primary Leydig cells provide some limitations as a model for quantification of gonadotropins.

Murine Leydig cells are naturally expressing mLhr sharing approximately 80% amino acid sequence identity with LHCGR at the hinge region, which is responsible of LH/hCG different cell signaling activation (Table 1). In fact, there is a specific, distinct site of interaction located within this region of the human receptor [15, 56, 57]. We could speculate that the mouse-specific amino acid sequence at the hinge region is not able to qualitatively discriminate between LH and hCG, reflecting the evolutionary differences embedded into the primate-specific LH and hCG dual ligand system. However, both human gonadotropins retain binding capability to the murine receptor [10]. Especially, the two human gonadotropins have similar quantitative binding to, but different affinities for mLhr [58]. This is due to low dissociation rate of the bound hCG, which contributes to its higher bioactivity than LH and may explain the quantitatively different cAMP recruitment elicited in vitro, demonstrated by our experiments. However, in the mouse Leydig tumor cells mLTC-1, both the human LH and hCG exhibit different reversibility of cAMP activation than rat and other mammals’ LHs [21], suggesting that rodent Leydig cells do not provide a system properly exploitable for human gonadotropin characterization, in spite of receptor binding.

### Knowledge by in vivo models

Previous studies demonstrated the PkA-dependence of pErk1/2 activation in mouse primary Leydig cells and tumor cell lines naturally expressing Lhr [59–62]. Moreover, the role of Erk1/2 in steroidogenesis and cell proliferation was extensively studied, both in vitro and in vivo, revealing the importance of the Erk1/2 signaling for Leydig cell functioning and steroidogenesis [22, 59, 63–66]. However, a clear comparison between LH and hCG in Leydig cells was never performed. Interesting data were provided by transgenic mouse models overexpressing hCG, instead of physiological levels of murine LH. Exaggerated hCG-induced signaling was linked to infertility and morphological alterations, including Leydig cell adenomas, in genetically modified mice [67–69]. Similar results were obtained in LHCGR knock-in mice [70]. On the other hands, transgenic mice overexpressing a chimeric LH containing the C-terminal peptide of hCG, resulting in extended hormone half-life with LH activity, leads to infertility, polycystic ovaries, and ovarian tumors [71]. Taken together, these data reveal the wide spectrum of effects mediated by Lhr at the physiological level, however, further efforts should be done to address the molecular mechanisms by which human gonadotropins exert their action through murine Lhr. These knowledge may be plausibly provided by in vitro studies.

## Conclusions

Our results demonstrated that human LH and hCG activate only quantitatively different, but qualitatively similar early signaling and equal downstream steroid synthesis in vitro in murine Leydig cells, raising concerns about the appropriateness of rodent in vivo bioassays evaluating LH/hCG-dependent endpoints. No different downstream LH/hCG-specific steroidogenic events are in contrast to what was observed in human primary granulosa cells, suggesting some limitations of the male mouse as a model for quantification of gonadotropins to be used for clinical purposes in human female. The functional diversity between human and rodent cell systems expressing orthologous receptors may affect the in vivo bioactivity of gonadotropins and reflects the evolutionary differences on reproductive endocrinology of these mammals. LH/hCG is a primate-specific ligand system acting on structurally and functionally different cells and receptor.

## Additional files

Additional file 1: Comparison between absolute levels of LH- and hCG-induced cAMP production by dose–response experiment. Murine primary Leydig cells were stimulated by increasing doses of hCG and LH, in the presence of 500 μM IBMX. Total cAMP was measured after 3 h of incubation. All the results are represented as means ± SEM in a logarithmic X-axis, then *non*-linear regressions were plotted (*n* = 4). (TIF 592 kb)
Additional file 2: Comparison between LH- and hCG-induced production of 17alpha-OH-progesterone measured by LC/MS. In order to confirm the reliability of testosterone levels detected by immunoassay (Fig. 4), the hormone was measured in 24 h-stimulated cells by EC_80_ LH and hCG, in the presence of 500 μM IBMX. Total steroid levels were measured by LC/MS and represented as means ± SEM (ng/ml; *n* = 2). (TIF 351 kb)
Additional file 3: Androstenedione levels measured by LC/MS, in LH- or hCG-stimulated Leydig cells. Androstenedione was measured in 24 h-stimulated cells by EC80 LH and hCG, in the presence of 500 μM IBMX. Hormone levels were means ± SEM (ng/ml; *n* = 2). (TIF 372 kb)
Additional file 4: Comparison between LH- and hCG-induced testosterone levels measured by LC/MS. Testosterone levels detected by immunoassay (Fig. 4) were confirmed using the LC/MS method. Hormone levels were measured in 24 h-stimulated cells by EC80 LH and hCG, in the presence of 500 μM IBMX. Total testosterone levels were represented as means ± SEM (ng/ml; *n* = 2). (TIF 347 kb)
Additional file 5: Alignment of LH receptor amino acid sequences obtained from the UniProt database (http://www.uniprot.org). *Homo sapiens* LHCGR (UniProt identifier: P22888), *Mus musculus* Lhr (P30730) and *Rattus norvegicus* Lhr (P16235) sequences were aligned by the UniProt online tool Clustal Omega 1.2.1 (http://www.uniprot.org/align). Boxes indicate sequence divergence; :=conservation of strong groups;. = conservation of weak groups or no consensus. (DOC 92 kb)

## Abbreviations

3βHSD: 3β-hydroxysteroid dehydrogenase
cAMP: Cyclic adenosine-monophosphate
CREB: cAMP-response element-binding protein
CTX: Cholera toxin
DHEA: Dehydroepiandrosterone
EC_50_: Half maximal effective concentrations
EC_80_: 80% effective dose
ERK1/2: Extracellular signal-regulated kinases 1 and 2
FSH: Follicle-stimulating hormone
GPCRs: G protein-coupled receptors
hCG: Human chorionic gonadotropin
Hprt: Mouse hypoxanthine phosphoribosyltransferase
IBMX: 3-isobutyl-1-methylxanthine
ICD: Intracellular domain
LC-MS: Liquid chromatography-mass spectrometry
LH: Luteinizing hormone
LHCGR: LH/hCG receptor
Mek: Mitogen-activated protein kinases
MEM: Minimal essential medium
mLhr: Murine LH receptor
MLTC-1: Mouse Leydig tumor cells
NCBI: National Center for Biotechnology Information
pCREB: Phospho-CREB
pERK1/2: Phospho-ERK1/2
PKA: Protein kinase A
rLhr: Rat LH receptor
STARD1: Steroidogenic acute regulatory protein
SVW: Vesicle weight gain
TMD: Transmembrane domain
