# Supplementary figures and images for: Human LH and hCG stimulate differently the early signalling pathways but result in equal testosterone synthesis in mouse Leydig cells in vitro
Source: Reprod Biol Endocrinol. 2017 Jan 5;15:2. doi: 10.1186/s12958-016-0224-3 (PMC5217336; doi:10.1186/s12958-016-0224-3)

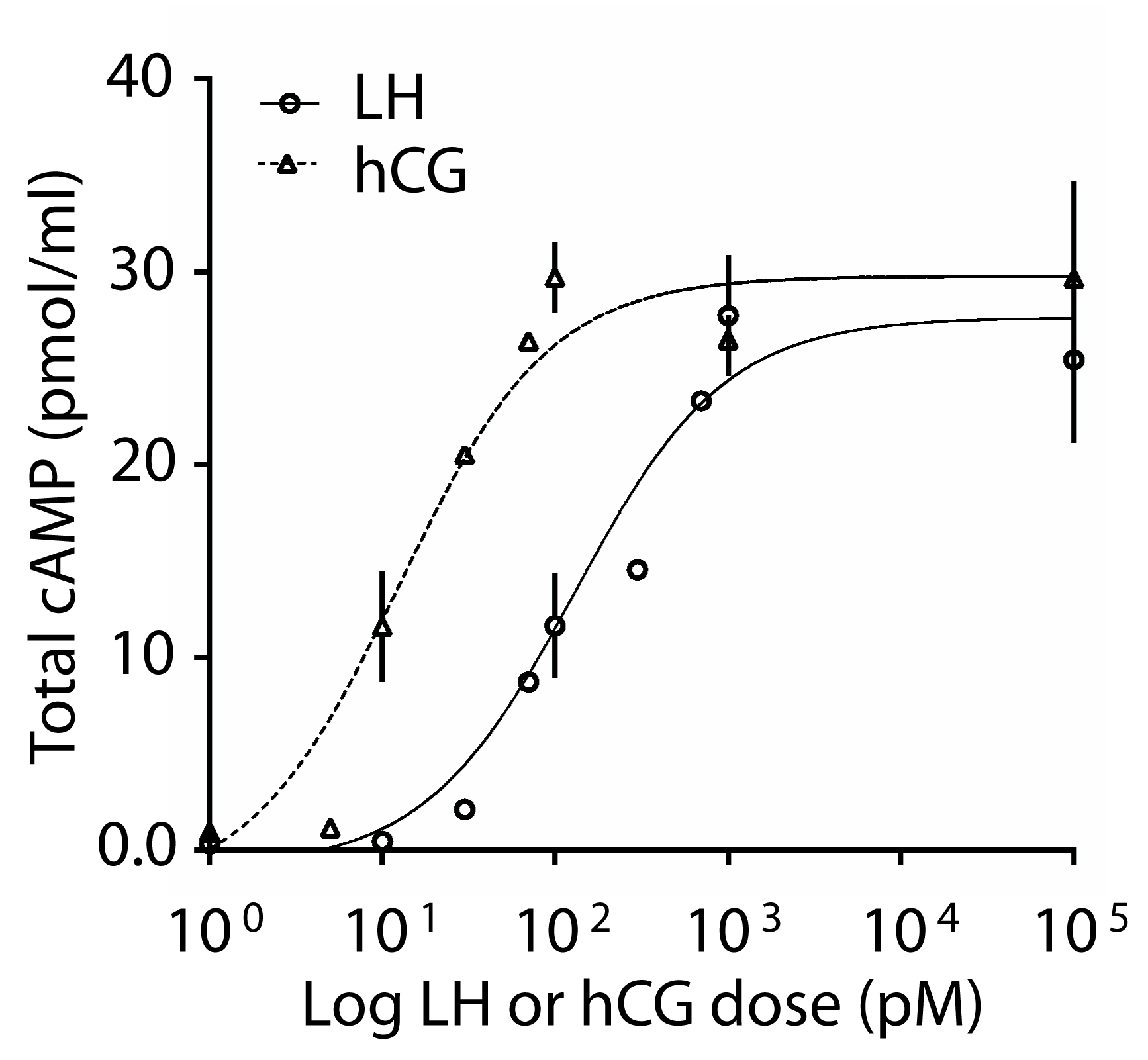

Supplement: Additional file 1: — Comparison between absolute levels of LH- and hCG-induced cAMP production by dose–response experiment. Murine primary Leydig cells were stimulated by increasing doses of hCG and LH, in the presence of 500 μM IBMX. Total cAMP was measured after 3 h of incubation. All the results are represented as means ± SEM in a logarithmic X-axis, then non-linear regressions were plotted (n = 4). (TIF 592 kb) [file 12958_2016_224_MOESM1_ESM.tif]

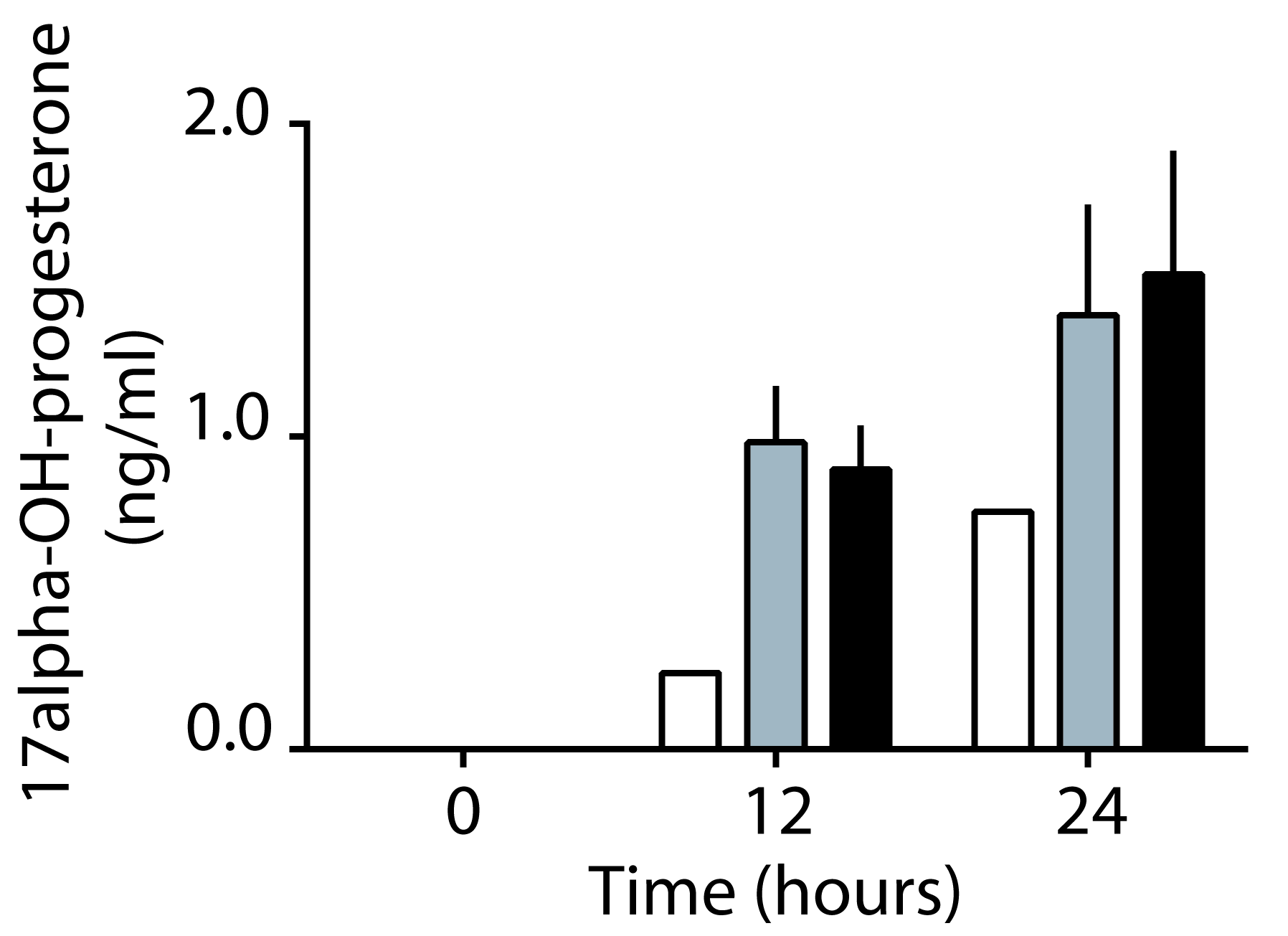

Supplement: Additional file 2: — Comparison between LH- and hCG-induced production of 17alpha-OH-progesterone measured by LC/MS. In order to confirm the reliability of testosterone levels detected by immunoassay (Fig. 4), the hormone was measured in 24 h-stimulated cells by EC80 LH and hCG, in the presence of 500 μM IBMX. Total steroid levels were measured by LC/MS and represented as means ± SEM (ng/ml; n = 2). (TIF 351 kb) [file 12958_2016_224_MOESM2_ESM.tif]

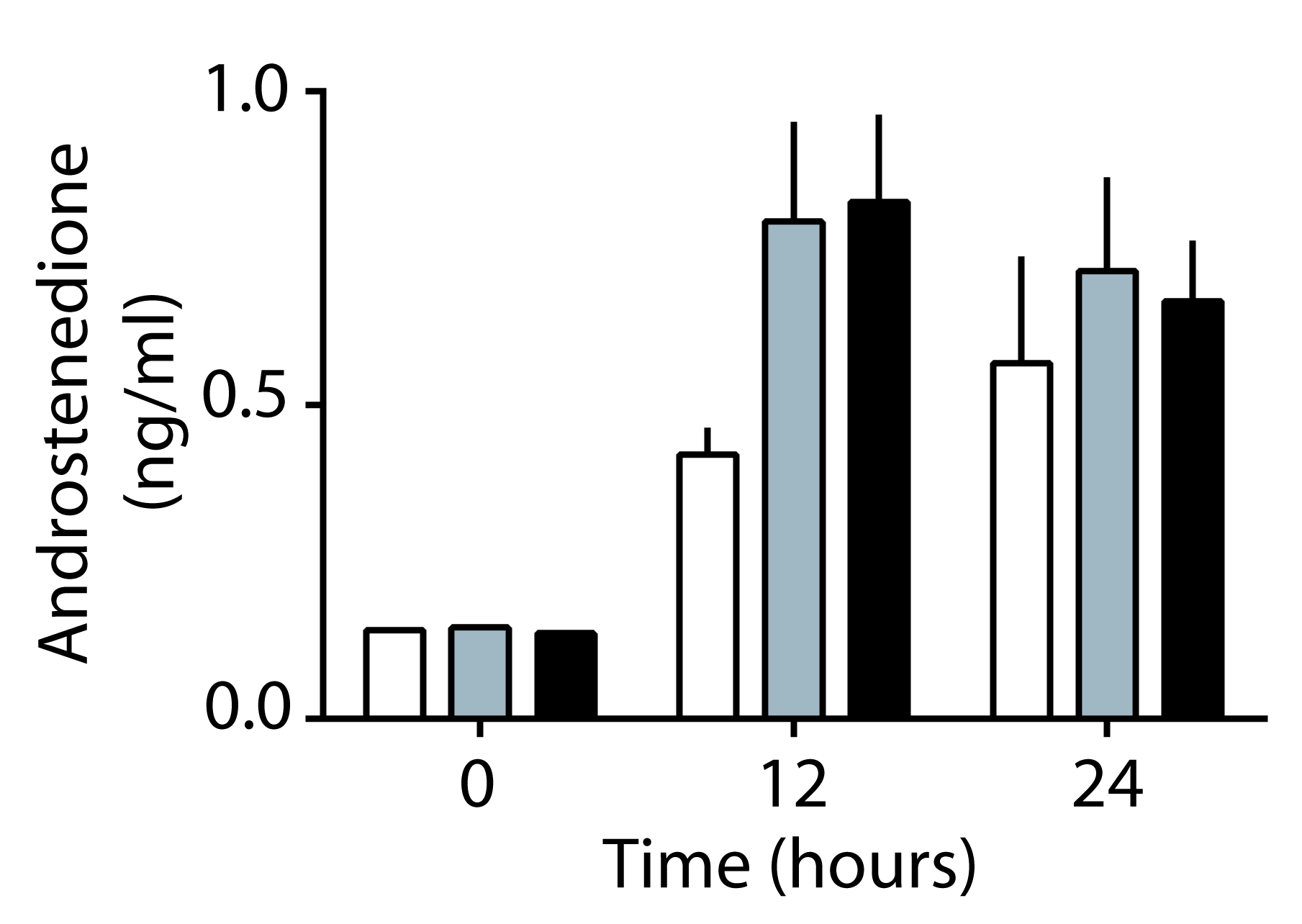

Supplement: Additional file 3: — Androstenedione levels measured by LC/MS, in LH- or hCG-stimulated Leydig cells. Androstenedione was measured in 24 h-stimulated cells by EC80 LH and hCG, in the presence of 500 μM IBMX. Hormone levels were means ± SEM (ng/ml; n = 2). (TIF 372 kb) [file 12958_2016_224_MOESM3_ESM.tif]

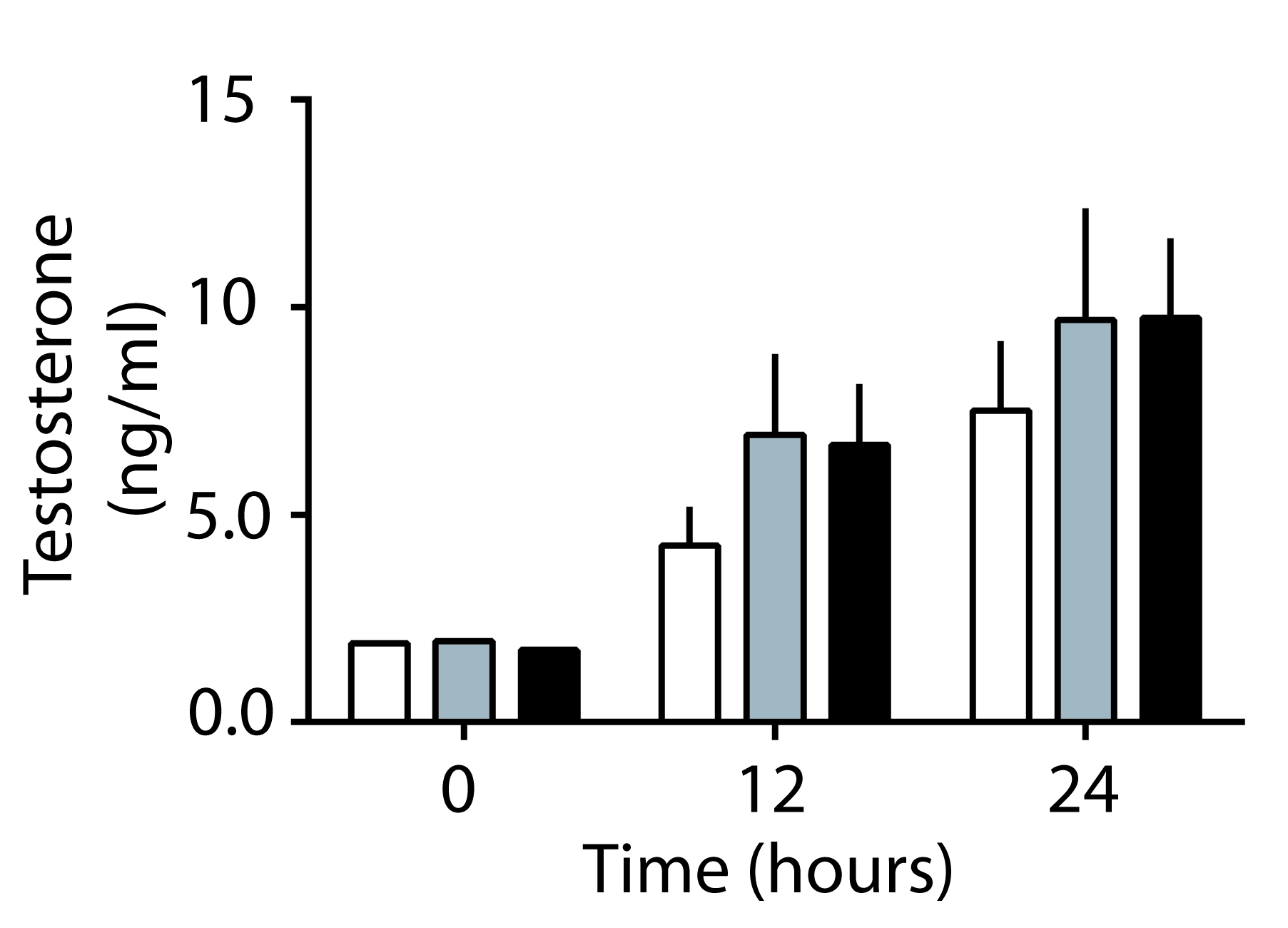

Supplement: Additional file 4: — Comparison between LH- and hCG-induced testosterone levels measured by LC/MS. Testosterone levels detected by immunoassay (Fig. 4) were confirmed using the LC/MS method. Hormone levels were measured in 24 h-stimulated cells by EC80 LH and hCG, in the presence of 500 μM IBMX. Total testosterone levels were represented as means ± SEM (ng/ml; n = 2). (TIF 347 kb) [file 12958_2016_224_MOESM4_ESM.tif]
