# Supplementary material for: Human LH and hCG stimulate differently the early signalling pathways but result in equal testosterone synthesis in mouse Leydig cells in vitro
Source: Reprod Biol Endocrinol. 2017 Jan 5;15:2. doi: 10.1186/s12958-016-0224-3 (PMC5217336; doi:10.1186/s12958-016-0224-3)
Supplement: Additional file 5: — Alignment of LH receptor amino acid sequences obtained from the UniProt database (http://www.uniprot.org). Homo sapiens LHCGR (UniProt identifier: P22888), Mus musculus Lhr (P30730) and Rattus norvegicus Lhr (P16235) sequences were aligned by the UniProt online tool Clustal Omega 1.2.1 (http://www.uniprot.org/align). Boxes indicate sequence divergence; :=conservation of strong groups;. = conservation of weak groups or no consensus. (DOC 92 kb) [file 12958_2016_224_MOESM5_ESM.doc]

**LHCGR / mLhr CLUSTAL O(1.2.1) partial sequence alignment**

Exon 9 Exon 10

**LHCGR**  LEATLTYPSHCCAFRNLPTKEQNFSHSI 296

**mLhr** LVATLTYPSHCCAFRNLPKKEQNFSFSI 300

Exon 10 Exon 11

. .

**LHCGR**  SENFSKQCESTVRKVNNKTLYSSMLAESELSGWDYEYGFCLPKTPRCAPEPDAFNPCEDI 356

**mLhr**  FENFSKQCESTVREANNETLYSAIFEENELSGWDYDYDFCSPKTLQCTPEPDAFNPCEDI 360

:. : ::: . : : :

TM1 IL1 TM2

**LHCGR**  MGYDFLRVLIWLINILAIMGNMTVLFVLLTSRYKLTVPRFLMCNLSFADFCMGLYLLLIA 416

**mLhr**  MGYAFLRVLIWLINILAIFGNLTVLFVLLTSRYKLTVPRFLMCNLSFADFCMGLYLLLIA 420

: :

EL1 TM3 IL2

**LHCGR**  SVDSQTKGQYYNHAIDWQTGSGCSTAGFFTVFASELSVYTLTVITLERWHTITYAIHLDQ 476

**mLhr**  SVDSQTKGQYYNHAIDWQTGSGCSAAGFFTVFASELSVYTLTVITLERWHTITYAVQLDQ 480

: ::

TM4 EL2

**LHCGR**  KLRLRHAILIMLGGWLFSSLIAMLPLVGVSNYMKVSICFPMDVETTLSQVYILTILILNV 536

**mLhr**  KLRLRHAIPIMLGGWIFSTLMATLPLVGVSSYMKVSICLPMDVESTLSQVYILSILLLNA 540

: : : . : : : :

TM5 IL3 TM6

**LHCGR** VAFFIICACYIKIYFAVRNPELMATNKDTKIAKKMAILIFTDFTCMAPISFFAISAAFKV 596

**mLhr**  VAFVVICACYVRIYFAVQNPELTAPNKDTKIAKKMAILIFTDFTCMAPISFFAISAAFKV 600

.: :: :

EL3 TM7

**LHCGR**  PLITVTNSKVLLVLFYPINSCANPFLYAIFTKTFQRDFFLLLSKFGCCKRRAELYRRKDF 656

**mLhr** PLITVTNSKVLLVLFYPVNSCANPFLYAVFTKAFQRDFFLLLSRFGCCKHRAELYRRKEF 660

: : : : : :

**LHCGR**  SAYTSNCKNGFTGSNKPSQSTLKLSTLHCQGTALLDKTRYTEC 699

**mLhr**  SACTFNSKNGFPRSSKPSQAALKLSIVHCQQPTPPRVLIQ--- 700

. . :: : :

**LHCGR / rLhr CLUSTAL O(1.2.1) partial sequence alignment**

Exon 9 Exon 10

**LHCGR**  LEATLTYPSHCCAFRNLPTKEQNFSHSI 296

**rLhr** LVATLTYPSHCCAFRNLPKKEQNFSFSI 300

. .

Exon 10 Exon 11

**LHCGR**  SENFSKQCESTVRKVNNKTLYSSMLAESELSGWDYEYGFCLPKTPRCAPEPDAFNPCEDI 356

**rLhr** FENFSKQCESTVRKADNETLYSAIFEENELSGWDYDYGFCSPKTLQCAPEPDAFNPCEDI 360

.: : ::: . : :

TM1 IL1 TM2

**LHCGR**  MGYDFLRVLIWLINILAIMGNMTVLFVLLTSRYKLTVPRFLMCNLSFADFCMGLYLLLIA 416

**rLhr** MGYAFLRVLIWLINILAIFGNLTVLFVLLTSRYKLTVPRFLMCNLSFADFCMGLYLLLIA 420

: :

EL1 TM3 IL2

**hLHCGR**  SVDSQTKGQYYNHAIDWQTGSGCSTAGFFTVFASELSVYTLTVITLERWHTITYAIHLDQ 476

**hLhr** SVDSQTKGQYYNHAIDWQTGSGCGAAGFFTVFASELSVYTLTVITLERWHTITYAVQLDQ 480

.: :

TM4 EL2

**LHCGR**  KLRLRHAILIMLGGWLFSSLIAMLPLVGVSNYMKVSICFPMDVETTLSQVYILTILILNV 536

**rLhr** KLRLRHAIPIMLGGWLFSTLIATMPLVGISNYMKVSICLPMDVESTLSQVYILSILILNV 540

: : : : : :

TM5 IL3 TM6

**LHCGR**  VAFFIICACYIKIYFAVRNPELMATNKDTKIAKKMAILIFTDFTCMAPISFFAISAAFKV 596

**rLhr** VAFVVICACYIRIYFAVQNPELTAPNKDTKIAKKMAILIFTDFTCMAPISFFAISAAFKV 600

.: : :

EL3 TM7

**LHCGR**  PLITVTNSKVLLVLFYPINSCANPFLYAIFTKTFQRDFFLLLSKFGCCKRRAELYRRKDF 656

**rLhr** PLITVTNSKILLVLFYPVNSCANPFLYAIFTKAFQRDFLLLLSRFGCCKRRAELYRRKEF 660

: : : : : :

**LHCGR**  SAYTSNCKNGFTGSNKPSQSTLKLSTLHCQGTALLDKTRYTEC 699

**rLhr** SAYTSNCKNGFPGASKPSQATLKLSTVHCQQPIPPRALTH--- 700

:. : : :

**mLhr / rLhr** CLUSTAL O(1.2.1) partial sequence alignment

Exon 9 Exon 10

**mLhr** LVATLTYPSHCCAFRNLPKKEQNFSFSI 300

Exon 10 Exon 11

**rLhr** LVATLTYPSHCCAFRNLPKKEQNFSFSI 300

**mLhr** FENFSKQCESTVREANNETLYSAIFEENELSGWDYDYDFCSPKTLQCTPEPDAFNPCEDI 360

**rLhr** FENFSKQCESTVRKADNETLYSAIFEENELSGWDYDYGFCSPKTLQCAPEPDAFNPCEDI 360

: : :

TM1 IL1 TM2

**mLhr**  MGYAFLRVLIWLINILAIFGNLTVLFVLLTSRYKLTVPRFLMCNLSFADFCMGLYLLLIA 420

**rLhr** MGYAFLRVLIWLINILAIFGNLTVLFVLLTSRYKLTVPRFLMCNLSFADFCMGLYLLLIA 420

EL1 TM3

**mLhr** SVDSQTKGQYYNHAIDWQTGSGCSAAGFFTVFASELSVYTLTVITLERWHTITYAVQLDQ 480

**rLhr** SVDSQTKGQYYNHAIDWQTGSGCGAAGFFTVFASELSVYTLTVITLERWHTITYAVQLDQ 480

.

IL2 TM4 EL2

**mLhr** KLRLRHAIPIMLGGWIFSTLMATLPLVGVSSYMKVSICLPMDVESTLSQVYILSILLLNA 540

**rLhr** KLRLRHAIPIMLGGWLFSTLIATMPLVGISNYMKVSICLPMDVESTLSQVYILSILILNV 540

: : : : . : .

TM5 IL3 TM6

**mLhr** VAFVVICACYVRIYFAVQNPELTAPNKDTKIAKKMAILIFTDFTCMAPISFFAISAAFKV 600

**rLhr** VAFVVICACYIRIYFAVQNPELTAPNKDTKIAKKMAILIFTDFTCMAPISFFAISAAFKV 600

:

EL3 TM7

**mLhr** PLITVTNSKVLLVLFYPVNSCANPFLYAVFTKAFQRDFFLLLSRFGCCKHRAELYRRKEF 660

**rLhr** PLITVTNSKILLVLFYPVNSCANPFLYAIFTKAFQRDFLLLLSRFGCCKRRAELYRRKEF 660

: : : :

**mLhr** SACTFNSKNGFPRSSKPSQAALKLSIVHCQQPTPPRVLIQ 700

**rLhr** SAYTSNCKNGFPGASKPSQATLKLSTVHCQQPIPPRALTH 700

. : : . :
